# Supplementary material for: Chromothripsis during telomere crisis is independent of NHEJ, and consistent with a replicative origin
Source: Genome Res. 2019 May;29(5):737–49. doi: 10.1101/gr.240705.118 (PMC6499312; doi:10.1101/gr.240705.118)
Supplement: Supplemental Material [file supp_gr.240705.118_Supplemental_file_1.zip › contigs/annotated_contigs/DB38/contig.2.DB38_length_374_mean_cov_23.0240641711.docx]

**DB38_length_374_mean_cov_23.0240641711**

```
CTCTCTCTCTCTCTCTCTCTGTCTCTCTCTGTCTCTCTCTCTCTCTC|TCTCTCTCTCTCTCTCTCTCTCTCTCT|CTCTCCCCTCCCT
 >chr22:25489704-25489778 + E=5e-30 p=0e+00     >chrUn_gl000220:120137-120464 + E=7e-18
CCCTCCTTGGTGCCTTCTCGGCTCGCTGCTGCTGCTGCCTCTGCCTCCACGGTTCAAGCAAACAGCAAGTTTTCTATTTCGAGTAAAGA
3
CGTAATTTCACCATTTTGGCCGGGCTGGTCTCGAACTCCCGACCTAGTGATCCGCCCGCCTCGGCCTCCCAAAGACTGCTGGGAGTACA

GATGTGAGCCACCATGCCCGGCCGATTCCTTCCTTTTTTCAATCTTATTTTCTGAACGCTGCCGTGTATGAACATACATCTACACATAC

ACACACACACACACACACAC
```
